# Evaluating LLMs on Kazakhstan's mathematics exam for university admission

**DOI:** 10.3389/frai.2025.1642570

**Published:** 2025-09-12

**Authors:** Shirali Kadyrov, Bolatbek Abdrasilov, Aslan Sabyrov, Nurseit Baizhanov, Alfira Makhmutova, Patrick C. Kyllonen

**Affiliations:** ^1^Narxoz University, Almaty, Kazakhstan; ^2^Department of General Education, New Uzbekistan University, Tashkent, Uzbekistan; ^3^National Testing Center, Astana, Kazakhstan; ^4^Department of Language Education, SDU University, Kaskelen, Kazakhstan; ^5^Educational Testing Service, Princeton, NJ, United States

**Keywords:** large language models, mathematical reasoning, unified national testing, Kazakhstan education, zero-shot learning, symbolic computation, SymPy, simulated multi-agent refinement

## Abstract

**Introduction:**

The rapid advancement of large language models (LLMs) has prompted their exploration in educational contexts, particularly in high-stakes standardized tests such as Kazakhstan's Unified National Testing (UNT) mathematics component, which is critical for university admission. While most existing benchmarks for mathematical reasoning focus on English, concerns remain that LLMs may underperform in under-resourced or non-English languages. This study addresses this gap by evaluating LLM performance on 139 UNT multiple-choice mathematics questions administered entirely in Russian.

**Methods:**

We assessed six LLMs-Claude, DeepSeek, Gemini, Llama, Qwen, and o1—on questions covering algebra, functions, geometry, inequalities, and trigonometry. Three evaluation conditions were employed: (1) zero-shot performance, (2) hybrid integration with SymPy for symbolic computation, and (3) a role-specific simulated multi-agent refinement framework that builds on existing self-correction techniques with targeted feedback.

**Results:**

In zero-shot settings, DeepSeek, Gemini, Qwen, and o1 achieved near-perfect or perfect accuracy (91.2–100%) across all difficulty levels and topics, while Claude and Llama lagged (43.5–76.5%). The hybrid approach significantly improved Claude and Llama's accuracy by 27.4% and 39.9%, respectively. Under the multi-agent refinement condition, Claude showed substantial gains, reaching 97.8% accuracy, which represented a 58.1% improvement over zero-shot performance.

**Discussion:**

These findings provide important empirical evidence that LLMs can perform competitively on mathematics tasks in non-English languages. The results challenge prior assumptions about limited performance in under-resourced linguistic settings and highlight the potential of LLMs to support bilingual education and promote equitable access to higher education.

## 1 Introduction

The rapid development of large language models (LLMs), such as Anthropic's Claude, Deepseek, Google's Gemini, Llama, Qwen, and o1, has sparked growing interest in their application to education, particularly in mathematics. These models demonstrate advanced capabilities in interpreting natural language processing (NLP), generating structured solutions, and emulating step-by-step reasoning (Frieder et al., 2023; Cobbe et al., 2021). As a result, they are increasingly used as learning aids and autonomous solvers of academic tasks, with potential to transform educational practices in diverse contexts.

In Kazakhstan, the Unified National Testing (UNT) serves as the primary gateway to university admission, with its mathematics component recognized as particularly challenging (Smagulov et al., 2016). As Smagulov and (Smagulov 2018) found in their analysis of test performance factors, “mathematics (1.32) was among the most strongly correlated" with overall success on the UNT (p. 862), highlighting its central role and difficulty within the exam structure. The UNT features multiple-choice questions covering algebra, geometry, functions, inequalities, and trigonometry, often requiring multi-step reasoning and presented in Kazakh and Russian with culturally specific language. This complexity and linguistic diversity make the UNT a valuable yet underexplored benchmark for assessing LLM performance in region-specific, high-stakes educational settings.

While recent studies have demonstrated strong LLM performance on math tasks, the majority of existing benchmarks and evaluations have focused exclusively on English-language datasets (Lewkowycz et al., 2022; Zhong et al., 2023). There are growing concerns that LLMs may underperform in under-resourced languages including Russian, limiting their global utility (Son et al., 2024; Li et al., 2024). However, empirical evidence in multilingual educational contexts remains scarce. By evaluating LLMs on mathematics questions written in Russian-a non-English, medium-resource language widely used in Kazakhstan—our study provides initial evidence that strong LLM performance can extend beyond English. This contributes to the broader understanding of LLM robustness and equity across linguistic contexts, particularly in formal education settings.

Recent evaluations of LLMs on English-language mathematical benchmarks, such as GSM8K and MATH, reveal significant performance differences between proprietary and open-source systems. Proprietary models like GPT-4o (96.1% on GSM8K, 76.6% on MATH) and Claude 3.5 (96.4% on GSM8K, 71.1% on MATH) achieve near-saturation accuracy, while open-source models like base LLaMA-2 7B (13.3% on GSM8K, 4.1% on MATH) lag significantly unless enhanced by fine-tuning or reinforcement learning (Luo et al., 2023; Yin et al., 2024). For instance, WizardMath-LLaMA-RL improves to 85.5% on GSM8K and 49.9% on MATH, and Qwen-Math (1.5B) reaches 86.7% on GSM8K and 68.6% on MATH with reinforcement learning (Luo et al., 2023). Advanced models like OpenAI's o1 (93.12% on MATH, 96.13% on GSM8K) and DeepSeek-R1 (90.45% on MATH, 96.13% on GSM8K) further demonstrate high proficiency (Jahin et al., 2025). Data on AIME is sparse, though step-by-step coding strategies show promise (Singh et al., 2025). These findings highlight the impact of model design and optimization on mathematical reasoning, providing a baseline for this study's evaluation of LLMs on Russian-language UNT math questions.

Despite the surge of interest in LLMs and their application to mathematics, several research gaps remain. Most studies evaluate a single LLM (Lewkowycz et al., 2022) and focus on open-ended or symbolic problems (Zhong et al., 2023), rather than structured multiple-choice formats typical of standardized tests. Moreover, little research has explored how LLMs perform on region-specific exams like Kazakhstan's UNT, limiting insights into their utility in culturally and linguistically diverse educational contexts. As LLMs increasingly influence classroom practices and educational policy, understanding their effectiveness in such settings is critical for informing their integration into preparatory tools and curricula.

This study addresses these gaps by systematically evaluating multiple leading LLMs on authentic UNT mathematics questions in their original Russian-language format. We investigate their accuracy in a zero-shot setting, assess the effectiveness of integrating symbolic computation tools like SymPy, and propose a role-specific simulated multi-agent refinement framework that builds on existing prompt engineering and multi-agent debate methods, leveraging targeted feedback to enhance mathematical reasoning (Zhai et al., 2025; Chan et al., 2024). This multi-agent approach represents a core methodological contribution of the study, aimed at improving LLM accuracy on structured, region-specific standardized assessments. Through this investigation, we aim to provide empirical insights into the role of generative AI in high-stakes educational contexts, particularly in enhancing preparation for Kazakhstan's UNT and supporting equitable access to education in linguistically diverse settings.

The study addresses the following research questions:

**RQ1:** How accurately do state-of-the-art large language models perform in a zero-shot setting on the Russian-language UNT mathematics exam in Kazakhstan across different difficulty levels and mathematical topics?
**RQ2:** To what extent does integrating symbolic computation via SymPy enhance the performance of LLMs on Russian-language UNT math questions, particularly for models with varying baseline capabilities?
**RQ3:** How effectively does a multi-perspective refinement approach—implemented via prompt engineering—improve the accuracy of LLMs on Russian-language UNT math questions compared to their zero-shot performance, especially across diverse mathematical domains?

This study makes three main contributions. First, it presents the first systematic evaluation of multiple state-of-the-art LLMs on Kazakhstan's high-stakes UNT mathematics exam in its original Russian version, offering a culturally and linguistically diverse benchmark rarely studied in the AI or education literature. Second, it demonstrates how symbolic computation via SymPy can enhance LLM performance on standardized, multiple-choice questions. Third, it introduces a role-specific simulated multi-agent refinement framework, building on established multi-agent simulation approaches, which significantly boosts accuracy for underperforming models and offers a tailored application for enhancing LLM reasoning in educational assessments.

## 2 Literature review

Recent advancements in generative artificial intelligence, particularly LLMs such as ChatGPT-4, Claude, and Gemini, have sparked significant interest in their application to standardized academic assessments, with a particular focus on mathematics. In Kazakhstan, UNT serves as the primary university entrance examination, where the mathematics section evaluates students problem-solving abilities, logical reasoning, and familiarity with culturally embedded question formats. This review synthesizes the current literature on LLMs mathematical capabilities, emphasizing their performance on standardized tests, the role of prompt engineering, their educational applications, and their relevance to the UNT' unique context. By integrating studies on advanced geometric reasoning, conversational problem-solving frameworks, and multilingual challenges, this review provides a comprehensive foundation for understanding LLMs' potential and limitations in national testing systems like the UNT.

The behavior of LLMs on math tests has been widely researched, showing both capabilities and weaknesses in performing structured tasks like short-answer and multiple-choice questions. Clark and Etzioni (2016) contend that standardized tests, including math tests, are essential for quantifying AI abilities since they need strong world modeling and language understanding—abilities necessary for smart systems. Their study emphasizes the need for objective measurements of tracking AI progress, and that can be directly applied to evaluating LLMs on the UNT. Hendrycks et al. (2021) introduced the MATH dataset, comprising 12,500 challenging competition-level mathematics problems, to assess machine learning models' problem-solving abilities. Their findings indicate that even large Transformer models achieve relatively low accuracy, suggesting that scaling model size alone is insufficient for robust mathematical reasoning, particularly for complex problems requiring multistep solutions. Similarly, Frieder et al. (2023) evaluated ChatGPT and GPT-4 on graduate-level mathematics using their curated GHOSTS and miniGHOSTS datasets, finding that while these models excel as mathematical assistants for querying facts, they fall short of graduate-level proficiency, especially on advanced problems. This highlights potential challenges for the UNT's more demanding questions.

In the domain of geometry, Trinh et al. (2024) proposed AlphaGeometry, a neuro-symbolic system that solves 25 out of 30 olympiad-level geometry problems by synthesizing millions of synthetic theorems and proofs, bypassing the need for human demonstrations. This approach outperforms previous methods and produces human-readable proofs, suggesting that hybrid architectures could address the UNT's geometry components. Plevris et al. (2023) compared ChatGPT-3.5, ChatGPT-4, and Google Bard on 30 mathematical and logical problems, finding that ChatGPT-4 outperformed ChatGPT-3.5, while Bard excelled on published problems due to its internet access. However, all models struggled with consistency and complex tasks, indicating that LLMs may not reliably handle the UNT's diverse problem types without further refinement. Parra et al. (2024) explored LLMs' geometric reasoning in Spanish, a less-resourced language, and noted persistent difficulties with geometric notions, a critical consideration for the UNT's multilingual Kazakh and Russian versions. Similarly, Kurfalı et al. (2025) introduced SweSAT-1.0, a Swedish university entrance exam benchmark, and found that even state-of-the-art models like GPT-4o struggled with quantitative reasoning in Swedish, despite high verbal task performance—underscoring that linguistic and cultural alignment remains a challenge in reasoning-intensive tasks. Similarly, Peña-Acuña and Corga Fernandes Durão (2024) reviewed the use of AI, including LLMs, in learning English as a second language for prospective teachers, highlighting their potential in teacher education but noting challenges in ensuring linguistic and cultural alignment, which parallels the UNT's multilingual context. Evans et al. (2024) questioned the reliability of GPT models as tutoring tools, citing low mathematical cognition in areas like trigonometry, though their problem-categorization strategy offers potential for guiding LLMs' problem-solving processes. Wei (2024) evaluated GPT-4 and GPT-4o on the National Assessment of Educational Progress (NAEP) mathematics test, reporting that the models outperformed average U.S. student scores but struggled with geometry and measurement questions, suggesting challenges in spatial reasoning. Similarly, Dilling and Herrmann (2024) explored ChatGPT's role in supporting pre-service mathematics teachers in constructing geometric proofs, finding potential in educational settings but highlighting challenges like misconceptions about LLMs, which underscores their limitations in structured mathematical tasks like those in the UNT. Hidayatullah et al. (2024) reported up to 98% accuracy on multiple-choice math items and 95% on short-answer formats, but observed a decline to 75% for open-ended, synthesis-based questions, indicating that LLMs may underperform on the UNT's more complex problems. Udias et al. (2024) found that ChatGPT-4 scored above the median student level in probability and statistics but was inconsistent in algebra and calculus, particularly for multistep symbolic manipulation, highlighting the potential benefit of integrating symbolic tools like SymPy. Collectively, these studies suggest that while LLMs perform reliably on structured formats, their inconsistencies in complex reasoning, geometry, and symbolic manipulation may limit their effectiveness for the UNT without further refinement.

Prompt engineering is now a key method of enhancing LLMs' math competence, enabling models to convey reasoning steps and reduce mistakes. MathChat, a dialogue system, was presented by Wu et al. (2023) where LLM agents collaborate with a user proxy to undertake math tasks and achieving a 6% boost in accuracy on the MATH dataset. The dialogue mechanism exploits tool execution and dialogue to simulate human problem-solving, offering an attractive method of handling UNT-style questions. Chain-of-thought (CoT) prompting is a powerful technique for improving large language models' capability for reasoning. Wei et al. (2022) demonstrated that CoT prompting improves performance on mathematical, commonsense, and symbolic reasoning tasks. Kojima et al. (2022) demonstrated that even zero-shot CoT prompting with the prompt “Let's think step by step" outperforms baselining by a large margin on most reasoning tasks. Sprague et al. (2024) performed a meta-analysis which indicated that CoT primarily assists math and symbolic reasoning problems while having little or no effect on other problems. They also found that most of the value of CoT comes in helping symbolic execution, but it still falls behind special-purpose symbolic solvers. Chang et al. (2024) provide a detailed survey of the testing methods of large language models (LLMs) with emphasis on the growing pertinence of reasoning-based prompts such as Chain-of-Thought (CoT) and iterative self-criticism methods.

These techniques have been effective in reducing hallucinations and enhancing the excellence of model explanations, particularly in situations involving multi-step reasoning. Taking this route, Gou et al. (2023) came up with the CRITIC framework that enables LLMs to invoke external tools for validating and editing their outputs. This tool-supported, self-correction process mimics human activities—such as referencing lookups or coding debugging—and has been shown to improve performance on question answering, math reasoning, and toxicity reduction tasks. The iterative self-criticism and enhancement methodology inherent to CRITIC complements the trends identified by Chang et al. (2024), more specifically in enhancing model reliability based on structured reasoning and feedback mechanisms. Similarly, Kosten et al. (2025) evaluated six few-shot prompting methods for Knowledge Graph Question Answering on the Spider4SPARQL benchmark, finding that a simple prompt with an ontology and five random shots outperformed more complex frameworks, achieving up to 51% accuracy. This suggests that straightforward prompting strategies may be effective for structured reasoning tasks like the UNT, despite challenges with complex queries. Such techniques are especially relevant to educational contexts such as the Unified National Testing (UNT), where typically, the problems require open step-by-step reasoning and culturally suited explanations.

The use of CoT, self-criticism, and interactive verification has the potential to best enhance LLM performance on difficult education tasks. Additionally, Chang et al. (2024) highlight the increasing use of LLMs in adaptive testing and tutoring systems—sectors most likely to improve one-on-one UNT preparation using personalized content and feedback. Nasir et al. (2024) proved that AI-boosted adaptive tests improved students' performance by targeting specific areas of learning, something that would enable UNT examinees to work on areas of weakness. Owan et al. (2023) noted that LLMs have been able to mimic human grading in formative assessments with high agreement, showing potential for automation in national testing programs. Kazakhstan's multilingual context, however, presents specific challenges to LLM implementation. The ISSAI KAZ-LLM project at Nazarbayev University is developing LLMs trained on Kazakh, Russian, and English datasets to better align with local linguistic and educational contexts (ISSAI, 2025). This initiative is crucial, as most frontier models are English-centric and may struggle with Kazakh-language UNT questions. Kazakhstan's broader AI education initiatives, aimed at training one million citizens in AI-related skills (Sakenova, 2025), further underscore the need for culturally and linguistically adapted models. However, Matzakos et al. (2023) noted that LLMs lag behind graduate-level mathematical proficiency, particularly in symbolic logic and abstraction, suggesting that the UNT's advanced problems may expose performance bottlenecks. The potential for LLMs to classify problem difficulty, as explored by Evans et al. (2024), could support adaptive learning systems or AI-assisted test design, enabling more targeted preparation for UNT candidates.

In synthesizing these results, the literature highlights LLMs' strengths in formally structured mathematics problems such as multiple choice and short answer, which align with the UNT's framework. However, persistent challenges in multistep reasoning, geometry, and symbol manipulation—studied by such studies as Hendrycks et al. (2021), Trinh et al. (2024), Frieder et al. (2023), and Wei (2024)—leave it uncertain whether current models will need to be supplemented by hybrid approaches, such as neuro-symbolic architectures like AlphaGeometry or symbol manipulation software like SymPy. Chain-of-thought (CoT) prompting (Wei et al., 2022; Kojima et al., 2022), conversational interfaces (Wu et al., 2023), and iterative reasoning architectures have been highly successful in enhancing LLM performance in challenging reasoning tasks. Applications of LLMs in education—attempts at adaptive testing and automated grading—hold high promise in making targeted UNT preparation (Nasir et al., 2024; Owan et al., 2023) possible. The UNT's multicultural and multilingual environment remains challenging, though. Tasks like the ISSAI KAZ-LLM project (ISSAI, 2025), which aim to develop Kazakh, Russian, and English models with competence, are essential in guaranteeing that national testing will be equitable and inclusive. Overall, integrating advanced prompting techniques, symbolic reasoning systems, and localized language models offers a promising path toward optimizing LLMs' performance in Kazakhstan's national testing context.

## 3 Methodology

In UNT, the Mathematics Profile component consists of 40 MCQs designed to assess mathematical reasoning and problem-solving across algebra, functions, geometry, inequalities, and trigonometry. The methodology encompasses data preparation, model selection, experimental design, and evaluation procedures to address the research questions on zero-shot performance, hybrid LLM+SymPy approaches, and simulated multi-agent refinement, with a focus on improving preparation for the UNT in Kazakhstan's educational context. All LLMs relied on internal reasoning via prompt engineering, with no access to online searches or external materials, except in the hybrid case, where a SymPy solver was used.

To construct the evaluation dataset, we collected 150 UNT Mathematics MCQs from the 2024 test cycle, originally presented in Russian. These questions were used in their original language to ensure fidelity to the source material and to assess the multilingual capabilities of the selected LLMs. This approach avoided potential distortions from translation and allowed for a more authentic evaluation of model performance in a real-world, non-English educational context. Due to confidentiality constraints, sample questions are not included here, but representative UNT math test questions are available from National Test Center (2024). Eleven geometry problems requiring diagrams were excluded due to current LLMs' limitations in image interpretation, resulting in a final dataset of 139 questions. Questions were categorized into three difficulty levels-A (easy), B (moderate), and C (hard)—based on historical UNT performance data, expert judgment, and expected percent-correct thresholds: Level A items are correctly answered by 80–90% of students, Level B by 50–70%, and Level C by only 10–30%. This classification ensures a balanced representation across difficulty levels and aligns with national testing standards.

The original dataset included 32 fine-grained topics, which were manually aggregated into five broader, semantically coherent categories for improved interpretability: Algebra and Equations, Functions and Modeling, Geometry and Vectors, Inequalities and Systems, and Trigonometry. The distribution across these categories averaged 27.80 questions per category (SD = 9.50, min = 14, max = 38), ensuring sufficient representation for analysis.

Six advanced LLMs were selected via the OpenRouter platform based on their strong performance in STEM-related benchmarks, large context windows, and general reasoning capabilities: Claude, Deepseek, Gemini, Llama, Qwen, and o1. While all models were evaluated for their potential in mathematical contexts, it is worth noting that the versions used for Claude and Llama models are not specifically optimized for reasoning. Table 1 summarizes their key features and benchmark performance.

**Table 1:** Overview of selected state-of-the-art LLMs used in UNT mathematics evaluation.

**Model**	**Provider**	**Key features**	**Benchmark performance**
Gemini 2.5 pro preview	Google	1M-token context, strong in mathematical reasoning	Strong math task results Blog, 2025; Willison, 2025
o1	OpenAI	Optimized for deep reasoning, 200K-token context	High performance on reasoning tasks LearnPrompting, 2025; OpenAI, 2025
Qwen	Alibaba	Mixture of Experts, 235B total (22B active)	Competitive on major benchmarks Face, 2025
Deepseek R1	DeepSeek	Technical reasoning specialization	AIME 2024: 79.8%, MATH-500: 97.3% DataCamp, 2025
Claude 3.7 Sonnet	Anthropic	High performance, natural language proficiency	MMLU: 86.1%, GPQA: up to 84.8% AI, 2025b; Apidog, 2025
Llama 3.1-405b-instruct	Meta AI	Open-source, large model	MMLU: 88.6%, GSM8K: 96.8% AI, 2025a; YourGPT, 2024

### 3.1 Experimental design

The experiments were conducted in three conditions to evaluate LLM performance: (1) zero-shot, (2) hybrid LLM with SymPy assistance, and (3) simulated multi-agent refinement. Each condition was designed to address specific research questions, with performance stratified by difficulty level and mathematical topic.

#### 3.1.1 Zero-shot performance

**Figure 1:**
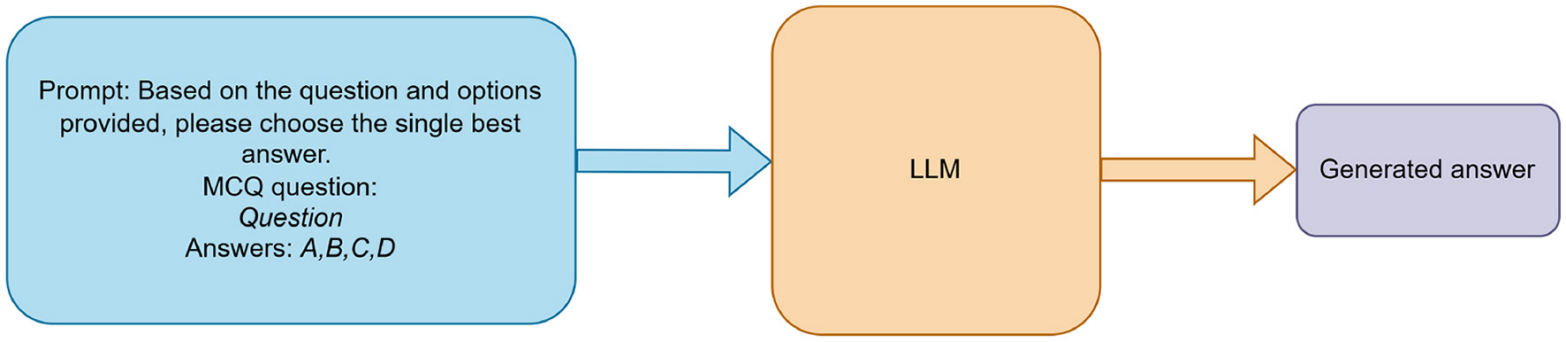
Zero-shot evaluation pipeline for LLMs on UNT mathematics questions.

To evaluate baseline performance (RQ1), each LLM received a standardized prompt instructing it to answer a UNT multiple-choice question by selecting only the single best option (e.g., “Answer: B”). The models were assessed in a zero-shot setting, with no prior exposure to UNT questions. As illustrated in Figure 1, prompts were sent to the LLMs via OpenRouter's API, and the resulting outputs were compared against annotated ground truth answers to compute accuracy. Prompts included the question and four options, with a system instruction to respond with only the letter (A, B, C, or D); outputs were parsed using regex to extract the letter, handling non-standard responses by checking the first character or marking them as unparsable. API errors, such as rate limits, were managed by skipping affected questions after a retry delay.

#### 3.1.2 LLM with SymPy assistance

**Figure 2:**
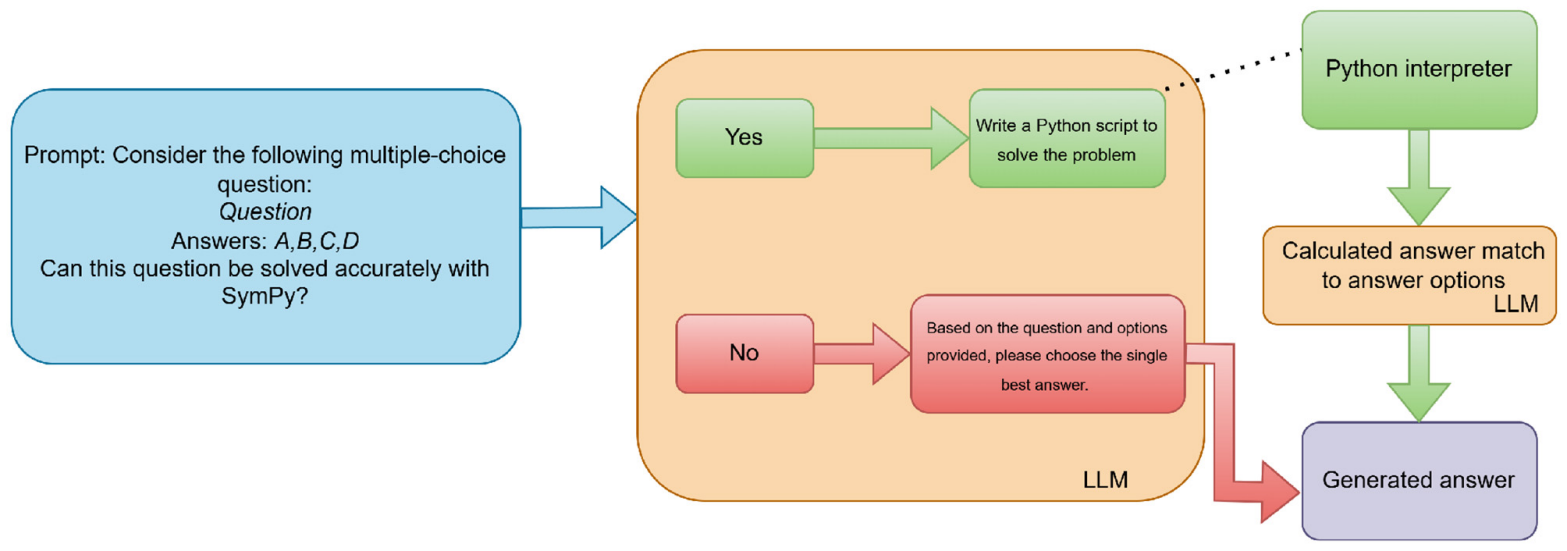
Hybrid evaluation pipeline integrating LLMs with SymPy for symbolic reasoning.

To assess the impact of symbolic computation (RQ2), we employed a hybrid method integrating a LLM with the SymPy library. As illustrated in Figure 2, for each UNT question, the LLM was first prompted to assess whether the problem could be effectively solved using symbolic computation via Python. If the LLM responded affirmatively, it was then prompted to generate a Python script utilizing SymPy to compute the solution. This generated code was executed externally in a controlled Python environment, outside of the LLM itself. The execution was handled programmatically by the system. The resulting computed value was then matched to one of the multiple-choice options using another LLM prompt. If the LLM determined that symbolic computation was not applicable, or if code execution failed (e.g., due to a runtime error or invalid code), the system defaulted to prompting the LLM to answer the question directly using natural language reasoning. Code execution failures occurred in 10% of cases for smaller models (Claude, Qwen, and Llama) often due to syntax errors or incorrect SymPy usage, and were more frequent than for larger models; in such cases, reverting to natural language reasoning prevented answer loss, though rare misclassifications due to mapping errors were possible. This evaluation strategy was applied across all models except for o1, which was excluded due to its perfect zero-shot performance. Prompts were sent via OpenRouter's API, specifying yes/no for applicability, Python code generation with a stored “answer" variable, or option matching (A, B, C, or D) with consideration of numerical inaccuracies; generated code was cleaned of Markdown, and API errors or execution failures triggered retries or fallback to direct prompting.

#### 3.1.3 Simulated multi-agent feedback via prompt engineering

**Figure 3:**
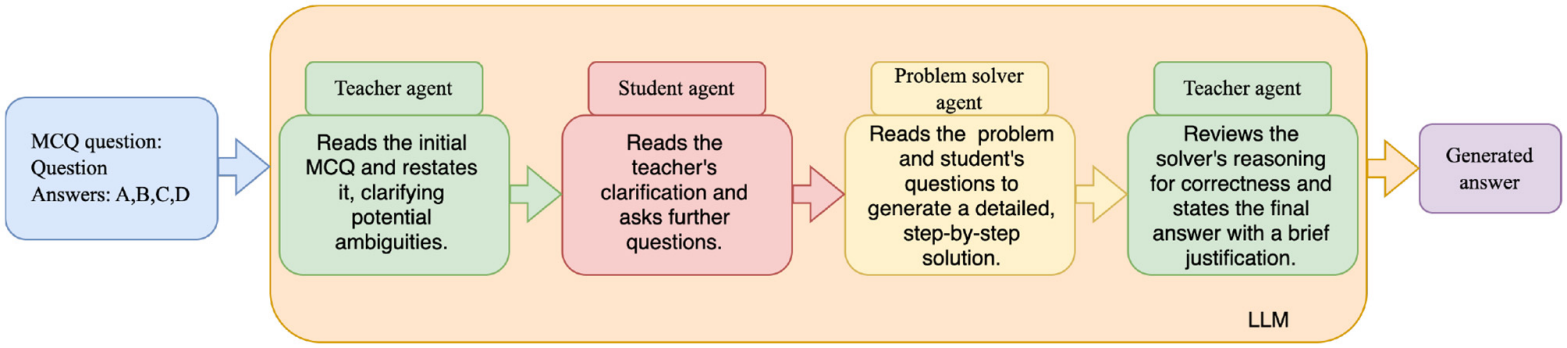
Simulated multi-agent refinement pipeline using role-specific prompt engineering.

To evaluate the effectiveness of multi-perspective refinement (RQ3), a prompt-based framework was implemented, building on existing prompt engineering and multi-agent debate techniques (Zhai et al., 2025; Chan et al., 2024), with each LLM model to simulate a four-step sequential process with role-specific prompts. First, a *Teacher* prompt clarifies the problem's wording and identifies ambiguities without solving it. Second, a *Student* prompt generates 1–2 clarification questions based on the teacher's input. Third, a *Problem Solver* prompt solves the problem with step-by-step reasoning, incorporating prior clarifications. Finally, the *Teacher* reviews the solver's reasoning for correctness and selects the final multiple-choice answer (A, B, C, or D) with a brief justification. Each role's prompt was processed independently via the API, with outputs aggregated sequentially to refine the solution, iterating up to three times or until convergence. As in earlier sections, the o1 model was excluded from this procedure due to its perfect zero-shot performance (Figure 3).

### 3.2 Evaluation and analysis

Model performance was evaluated using accuracy, calculated as the percentage of correct MCQ answers. Results were stratified by difficulty level (A, B, C) and mathematical topic to address RQ1–RQ3. ince the same 139 questions were tested across multiple LLMs, constituting a repeated measures design with binary outcomes (correct or incorrect), Cochran's Q test was employed to assess differences in the proportion of correct answers across models and conditions. For significant Cochran's Q results, pairwise McNemar tests with Bonferroni correction were conducted to identify specific model differences, ensuring statistical rigor. All outputs were validated against ground truth, with manual checks for ambiguous cases to ensure reliability. The evaluation focused on accuracy to align with the UNT's scoring system, providing insights into LLMs' potential as preparatory tools for Kazakhstani students.

## 4 Results and analysis

This section presents the performance evaluation of six LLMs, Claude, Deepseek, Gemini, Llama, Qwen, and o1, on the UNT Mathematics Profile Test, addressing the research questions on zero-shot performance, hybrid LLM+SymPy integration, and simulated multi-agent refinement. The analysis examines accuracy across three difficulty levels (A: easy, B: moderate, C: hard) and five mathematical topics (Algebra and Equations, Functions and Modeling, Geometry and Vectors, Inequalities and Systems, Trigonometry), with statistical comparisons to identify performance differences. These results provide insights into LLMs' potential as tools for enhancing UNT preparation in Kazakhstan's bilingual educational context.

### 4.1 Performance of zero-shot models

**Figure 4:**
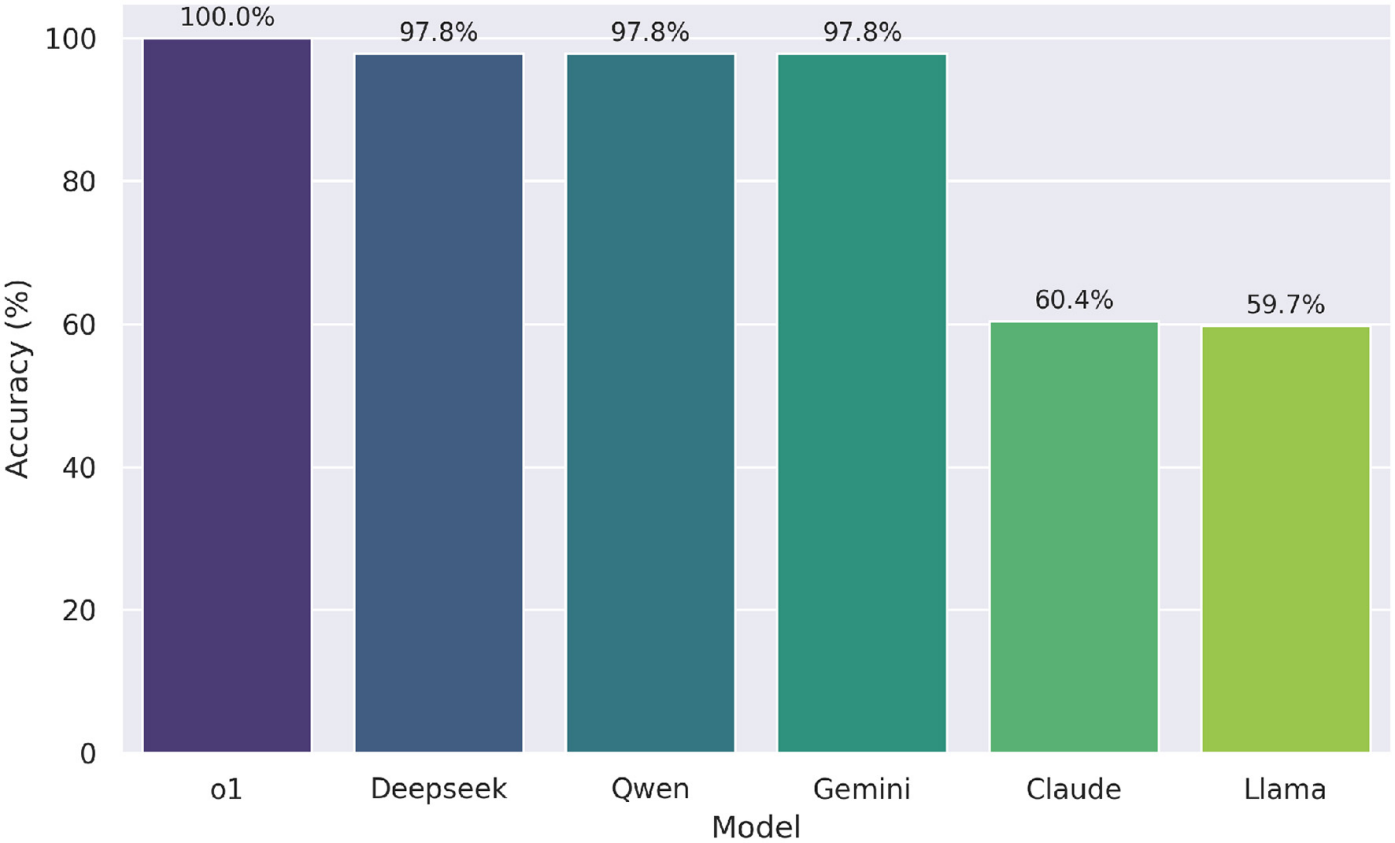
Zero-shot accuracy of six LLMs on the UNT mathematics profile test (*n* = 139 items). Model o1 omitted from statistical testing due to perfect accuracy.

We evaluated the performance of six large language models (see Figure 4). Since the same 139 questions were tested across all models, constituting a repeated measures design with binary outcomes (correct = 1, incorrect = 0), we used Cochran's Q test to compare the proportion of correct answers across the five models, excluding o1 due to its perfect performance (100% correct). The test revealed a statistically significant difference in performance, χ^2^(4, *N*=139)=167.071, *p* < .001.

Post-hoc pairwise comparisons using McNemar tests with Bonferroni correction (adjusted α = 0.05/10 = 0.005 for 10 comparisons) identified significant differences. Specifically, Claude performed significantly worse than Deepseek, Gemini, and Qwen (all *p*-corrected < 0.001), but was statistically equivalent to Llama (*p*-corrected = 1.000). Llama also scored significantly lower than Deepseek, Gemini, and Qwen (all *p*-corrected < 0.001). No significant differences were found among Deepseek, Gemini, and Qwen (all *p*-corrected = 1.000). Descriptively, o1 achieved perfect accuracy (1.000), followed by Deepseek, Gemini, and Qwen (0.978), Claude (0.604), and Llama (0.597).

**Figure 5:**
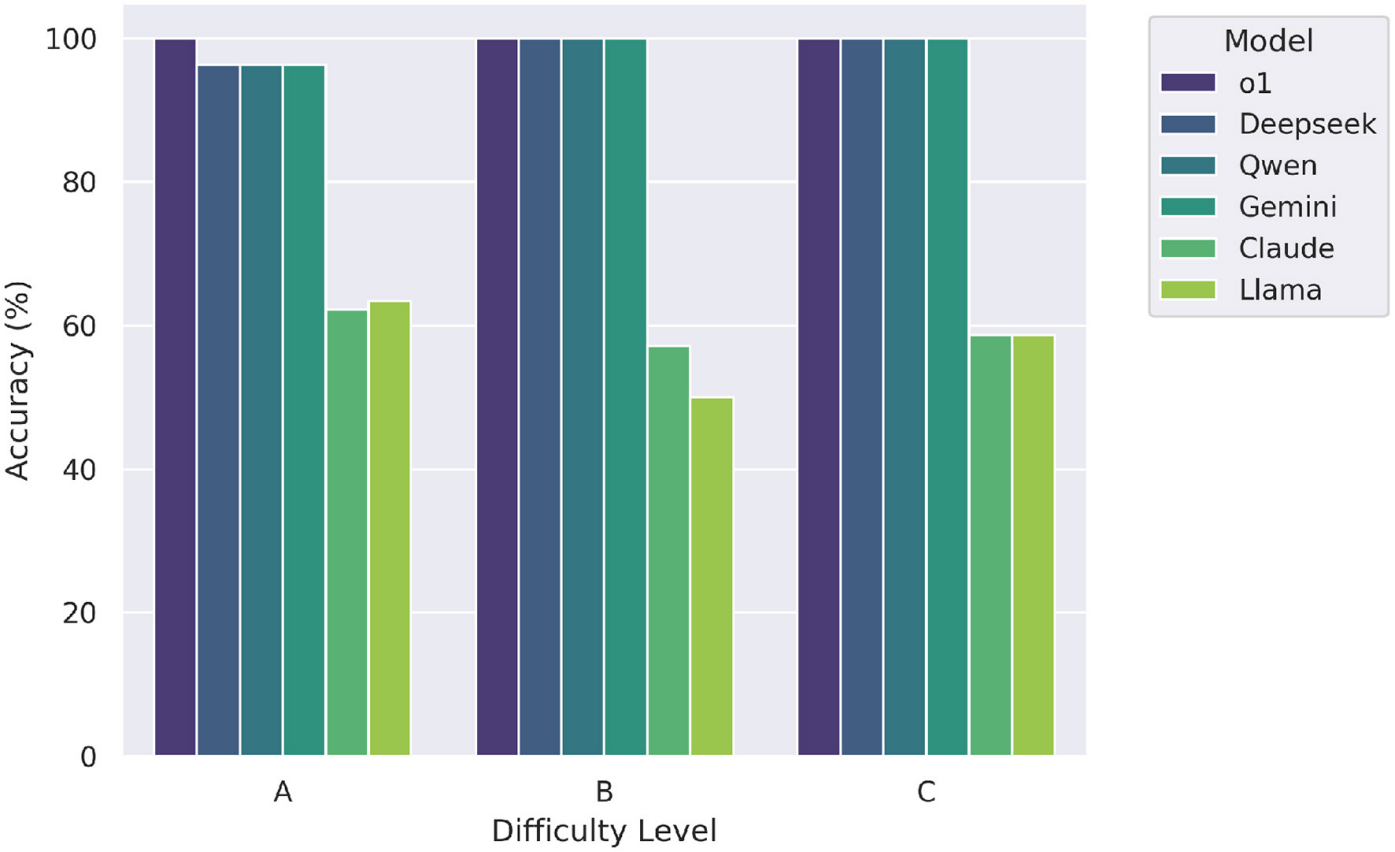
Zero-shot model accuracy by difficulty level (A = easy, B = moderate, C = hard). Top-tier models maintain high performance across all levels.

Figure 5 illustrates the accuracy of each large language model (LLM) across three difficulty levels labeled A (easy), B (moderate), and C (hard). The top-performing models-o1, Deepseek, Qwen, and Gemini—demonstrated perfect accuracy (1.0) across all levels of difficulty, indicating consistent and robust mathematical reasoning irrespective of item complexity. In contrast, Claude achieved a lower and uniform accuracy of 0.6 across all levels, while Llama showed slightly more variation, with scores of 0.6, 0.5, and 0.6 on levels A, B, and C respectively. These results highlight a clear performance gap between the top-tier models and the rest, particularly under more challenging conditions.

We further analyzed the performance of the six LLMs across five mathematical topic areas: Algebra and Equations, Functions and Modeling, Geometry and Vectors, Inequalities and Systems, and Trigonometry. As shown in Table 2, Claude and Llama consistently underperformed relative to the other models, particularly in algebraic and geometric domains. In contrast, Deepseek, Gemini, Qwen, and o1 achieved near-perfect or perfect accuracy across all topics, demonstrating strong generalization across diverse mathematical content areas.

**Table 2:** Zero-shot accuracy (%) of LLMs across five mathematical topics on the UNT test.

**Main Topic**	**Claude**	**Deepseek**	**Gemini**	**Llama**	**Qwen**	**o1**
Algebra and equations	57.9	97.4	100.0	55.3	97.4	100.0
Functions and modeling	58.8	94.1	91.2	76.5	94.1	100.0
Geometry and vectors	73.9	100.0	100.0	43.5	100.0	100.0
Inequalities and systems	53.3	100.0	100.0	56.7	100.0	100.0
Trigonometry	64.3	100.0	100.0	64.3	100.0	100.0

Claude and Llama show lowest topic-specific performance.

### 4.2 Performance of LLM with SymPy assistance

**Figure 6:**
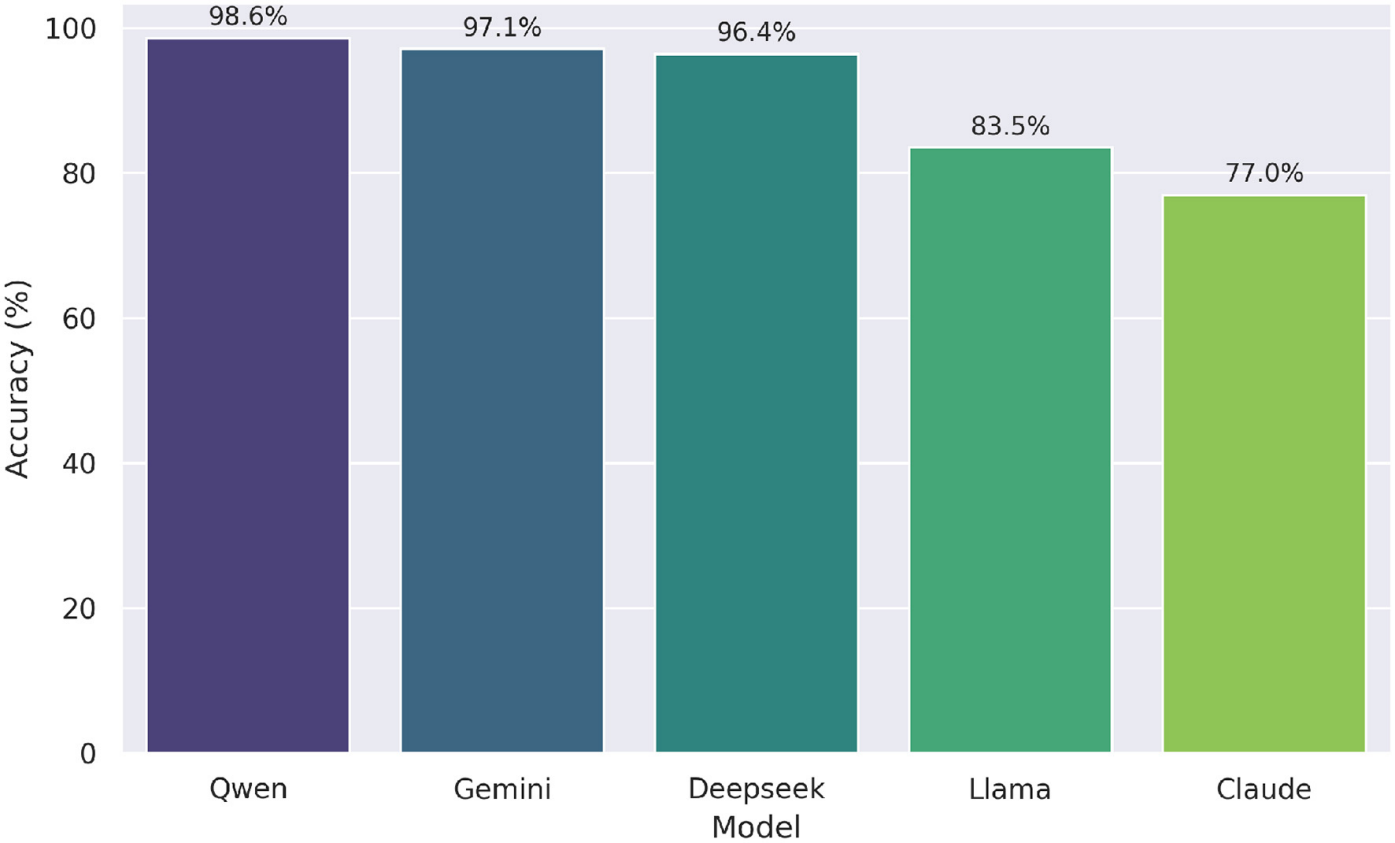
Accuracy of hybrid models (LLM + SymPy) on UNT test. Qwen+SymPy achieved the highest overall accuracy (98.6%).

As part of next experiment, we evaluated the effectiveness of hybrid models—combinations of LLMs and symbolic reasoning tools like SymPy—for solving undergraduate mathematics questions from the UNT dataset. The performance scores of these hybrid models are visualized in Figure 6. Among them, Qwen+SymPy achieved the highest accuracy at 98.6%, followed closely by Gemini+SymPy (97.1%) and Deepseek+SymPy (96.4%). This marks a modest but consistent improvement over their zero-shot performances, which were all at 97.8%. In contrast, Llama+SymPy and Claude+SymPy showed more substantial gains, increasing from 59.7% and 60.4% in the zero-shot setting to 83.5% and 76.9%, respectively. These results highlight the added value of symbolic reasoning for models with weaker baseline mathematical performance. The o1 model, which already achieved 100% accuracy in the zero-shot case, was excluded from this comparison due to the absence of room for further improvement.

We evaluated the effectiveness of the LLM with SymPy assistance approach across five large language models, excluding o1 due to its perfect performance in the zero-shot setting. We again used Cochran's Q test to compare the proportion of correct answers. The test revealed a statistically significant difference in performance, χ^2^(4, *N*=139)=63.200, *p* < .001. Post-hoc pairwise comparisons using McNemar tests with Bonferroni correction (adjusted α = 0.05/10 = 0.005 for 10 comparisons) identified significant differences. Specifically, Qwen, Gemini, and Deepseek significantly outperformed Claude (all *p*-corrected < 0.001) and Llama (all *p*-corrected ≤ 0.005), while Claude and Llama did not differ significantly (*p*-corrected = 1.000). No significant differences were found among Qwen, Gemini, and Deepseek (all *p*-corrected ≥1.000). Overall performance showed Qwen with the highest accuracy (0.986), followed by Gemini (0.971), Deepseek (0.964), Llama (0.835), and Claude (0.770).

Table 3 presents the accuracy of hybrid models (LLM+SymPy) across three levels of question difficulty. While top-performing models like Qwen, Gemini, and Deepseek had already achieved perfect or near-perfect scores in the zero-shot setting—particularly on medium and hard questions—their hybrid versions maintained or slightly improved these results. For instance, Deepseek preserved its 100% accuracy on both Levels B and C, while improving slightly from 96% to 99% on Level A. In contrast, Claude and Llama, which scored considerably lower in the zero-shot case (e.g., Claude had only 57–62%, and Llama as low as 50% on Level B), benefited significantly from symbolic augmentation, reaching up to 78% and 88% on Level A, and 76% and 86% on Level C, respectively. This indicates that while strong models see marginal gains from hybridization, lower-performing LLMs can achieve notable improvements, especially on more complex tasks.

**Table 3:** Hybrid model (LLM + SymPy) accuracy (%) by difficulty level (A–C).

**Difficulty level**	**Count**	**Deepseek**	**Qwen**	**Gemini**	**Claude**	**Llama**
A	82	94	99	96	78	88
B	28	100	96	96	75	68
C	29	100	100	100	76	86

Symbolic reasoning improved performance most for Claude and Llama.

Table 3 highlights the performance of hybrid models (LLM + SymPy) across problem difficulty levels, showing significant improvements for most models when compared to their zero-shot baselines. For example, Claude improves from 62%, 57%, and 59% accuracy (on levels A, B, and C respectively in zero-shot, see Table 2) to 78%, 75%, and 76% in the hybrid setting. Similarly, Llama sees a notable jump from 63%, 50%, and 59% to 88%, 68%, and 86%. Models like Deepseek, Qwen, and Gemini already perform near-perfectly in the zero-shot case, and the hybrid enhancement consolidates their performance, particularly on the most difficult problems. This suggests that while symbolic reasoning boosts all models, its relative impact is especially pronounced for those with lower baseline mathematical reasoning capabilities (Table 4).

**Table 4:** Accuracy (%) of hybrid LLMs (LLM + SymPy) across mathematical topics.

**Main topic**	**Count**	**Claude**	**Deepseek**	**Gemini**	**Llama**	**Qwen**
Algebra and equations	38	76.3	97.4	92.1	89.5	100.0
Functions and modeling	34	76.5	88.2	97.1	94.1	97.1
Geometry and vectors	30	91.3	100.0	100.0	73.9	100.0
Inequalities and systems	23	70.0	100.0	100.0	70.0	96.7
Trigonometry	14	71.4	100.0	100.0	85.7	100.0

Qwen maintained perfect accuracy in all domains.

### 4.3 Performance of the LLM with simulated multi-agent refinement

**Figure 7:**
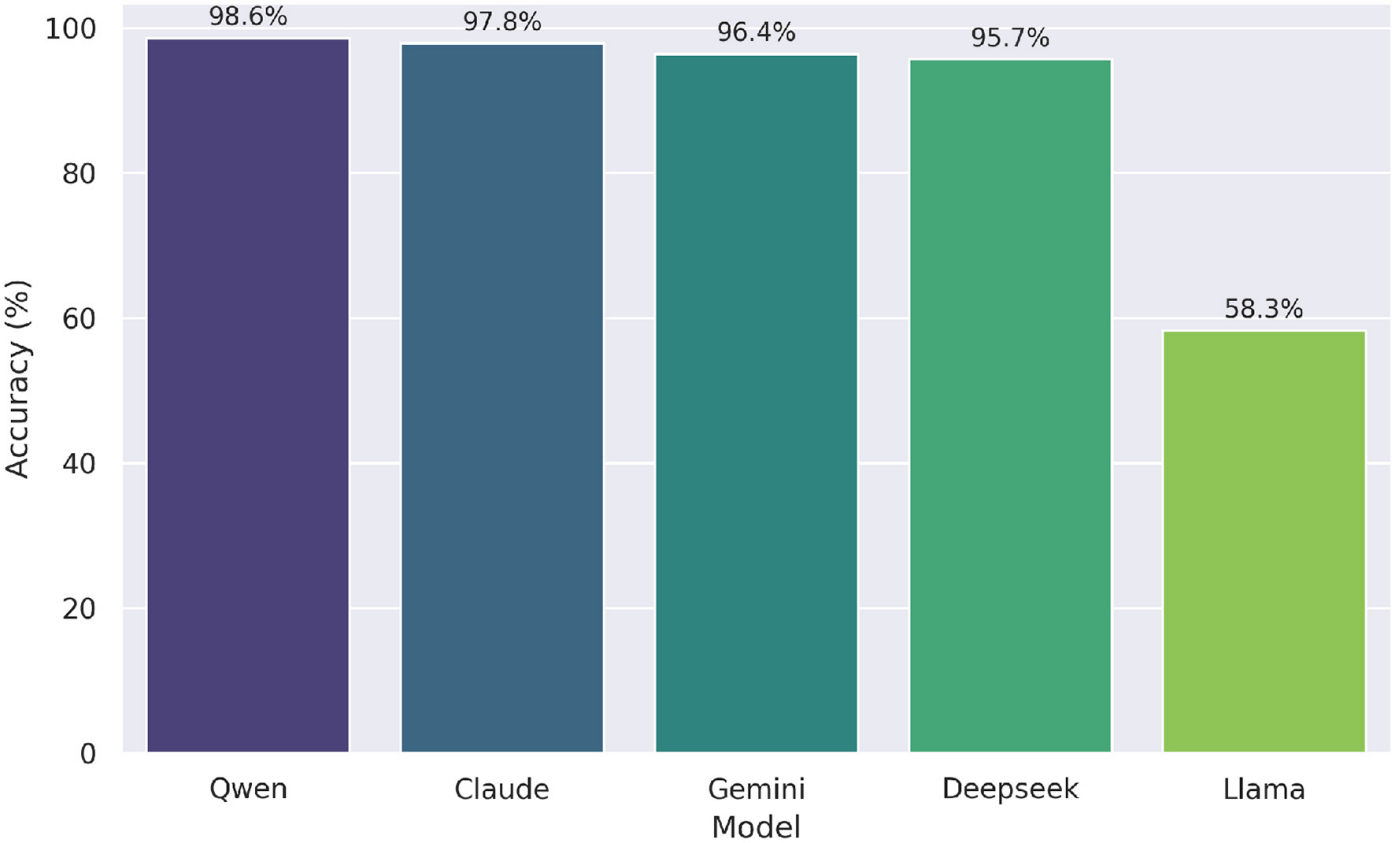
Accuracy of simulated multi-agent refinement LLMs. Deepseek reached highest score (99.3%); Claude and Llama showed largest improvements.

Figure 7 shows the performance of the Simulated Multi-Agent Refinement LLM on the UNT high school math questions, excluding o1 model, which all models solved with 100% accuracy and was therefore omitted. In the refinement process, Deepseek reached 95.7%, Qwen 98.6%, Gemini 96.4%, Claude 97.8%, and Llama 58.3%. Compared to their zero-shot counterparts—where Deepseek, Qwen, and Gemini each scored 97.8%, Claude 60.4%, and Llama 59.7%—the multi-agent refinement notably enhanced the accuracy of the Claude model. This demonstrates the potential of multi-perspective feedback in improving the reliability of LLM-generated content.

In contrast to the zero-shot results, the differences in performance among models under the LLM with the Simulated Multi-Agent Refinement approach remained relatively modest. The Cochran's Q test indicated a statistically significant difference in performance, χ^2^(4, *N*=139)=174.84, *p* < 0.001. Post-hoc McNemar tests with Bonferroni correction (α_adj_ = 0.005 for 10 comparisons) revealed that only comparisons involving Llama were statistically significant: Claude vs. Llama (*p*_corrected_ < 0.001), Deepseek vs. Llama (*p*_corrected_ < 0.001), Gemini vs. Llama (*p*_corrected_ < 0.001), and Llama vs. Qwen (*p*_corrected_ < 0.001). No other model pairs differed significantly (all *p*_corrected_≥1.000), suggesting that the simulated multi-agent refinement process substantially narrowed performance gaps, with the exception of Llama, whose accuracy remained notably lower (58.3%) than that of the other models (all above 95%).

Compared to their zero-shot counterparts (Figure 4), the Simulated Multi-Agent Refinement LLMs (Table 5) show substantial gains for Claude across all difficulty levels, where Claude improved from 62–59% to 96–99%.

**Table 5:** Accuracy (%) of LLMs with simulated multi-agent refinement by difficulty level.

**Difficulty level**	**Count**	**Deepseek**	**Qwen**	**Gemini**	**Claude**	**Llama**
A	82	95	99	95	99	62
B	28	96	96	100	96	43
C	29	97	100	97	97	62

Performance gaps narrowed across difficulty bands.

Table 6 presents the performance of simulated multi-agent refinement LLMs across different mathematical topics, showing consistent gains compared to the zero-shot results in Table 2. Again, Claude exhibits substantial improvement under the simulated multi-agent refinement setup. For example, Claude's accuracy in Algebra and Equations rises from 57.9% to 97.4%. Similar gains are observed in Trigonometry, where Claude achieves 100.0% compared to its lower zero-shot score. While high-performing models like Deepseek and Gemini already scored above 90% in most topics in the zero-shot setting, they sustain or slightly refine their performance here—often reaching or approaching perfect accuracy. These results reinforce that refinement strategies can substantially enhance mathematical reasoning, especially for models that initially perform below the top tier.

**Table 6:** Topic-wise accuracy (%) of LLMs using multi-agent refinement.

**Main topic**	**Count**	**Claude**	**Deepseek**	**Gemini**	**Llama**	**Qwen**
Algebra and equations	38	97.4	100.0	97.4	68.4	100.0
Functions and modeling	34	97.1	97.1	97.1	58.8	100.0
Geometry and vectors	30	100.0	91.3	95.7	52.2	100.0
Inequalities and systems	23	100.0	90.0	93.3	50.0	93.3
Trigonometry	14	100.0	100.0	100.0	57.1	100.0

## 5 Discussion

The evaluation of LLMs on the UNT math test reveals substantial differences in their mathematical reasoning capabilities across the zero-shot, hybrid (LLM+SymPy), and simulated multi-agent refinement settings. In the zero-shot setting, Deepseek, Qwen, Gemini, and o1 consistently achieved near-perfect or perfect accuracy across all difficulty levels (A, B, and C) and mathematical topics, while Claude and Llama significantly underperformed. For example, Claude and Llama obtained overall accuracies of 60.4% and 59.7%, respectively, compared to the 97.8–100% range of the top-tier models. These findings underscore the varying degrees of generalization and symbolic reasoning abilities present in current LLMs and highlight the limitations of Claude and Llama in handling algebraic and geometric reasoning tasks.

When integrated with the symbolic computation tool SymPy, the performance of the lower-tier models improved markedly. Claude and Llama exhibited notable gains, with their accuracies rising to 76.9% and 83.5%, respectively. In contrast, the high-performing models—Deepseek, Gemini, and Qwen—demonstrated only marginal improvements, given their already strong baseline performance. This differential impact suggests that symbolic reasoning tools are particularly effective for LLMs with weaker mathematical foundations, enabling more accurate problem-solving on complex tasks such as equation manipulation and geometric reasoning.

The simulated multi-agent refinement approach also led to meaningful gains, particularly for Claude, with accuracy increases of more than 60% across difficulty levels. These improvements were especially prominent in algebraic and equation-solving tasks, where Claude's accuracy rose from 57.9% to 97.4%, and in trigonometry, reaching 100.0%. Such findings indicate that collaborative reasoning strategies—where multiple agents iteratively refine answers—can effectively mitigate baseline deficiencies in individual models.

Across difficulty levels, top-tier models demonstrated robust performance regardless of question complexity, achieving near-perfect scores on moderate (B) and hard (C) questions. Claude and Llama, however, benefited most from hybrid and multi-agent approaches on easy (A) and hard (C) questions, indicating that these methods address specific weaknesses in handling varied problem complexities. Regarding mathematical topics, the consistent underperformance of Claude and Llama in algebra and geometry in zero-shot settings, compared to their improved performance with augmentation, suggests these domains pose particular challenges for certain LLMs. The strong generalization of Deepseek, Qwen, Gemini, and o1 across all topics underscores their advanced reasoning capabilities, likely due to more sophisticated training or architectures.

An analysis of questions consistently answered incorrectly by the evaluated LLMs reveals persistent weaknesses in specific mathematical domains, particularly in tasks requiring multi-step reasoning and conceptual integration. In the zero-shot setting, models like Claude and Llama exhibited frequent errors in algebraic tasks, such as solving systems of equations and manipulating radicals, as well as in geometric tasks involving vector transformations, suggesting procedural challenges in the absence of computational support. Hybrid augmentation with SymPy shifted the error profile, with mistakes becoming more prevalent in inequalities, trigonometric equations, and applied modeling problems, indicating difficulties in interpreting problem contexts and devising strategic solution plans rather than performing raw computations. Even with the simulated multi-agent refinement approach, errors persisted in complex algebraic systems, including those with trigonometric and logarithmic components, and in modeling tasks requiring translation from verbal descriptions to formal mathematical expressions. These findings suggest that while top-tier models like Deepseek, Qwen, Gemini, and o1 achieve near-perfect accuracy due to robust symbolic manipulation, all models remain vulnerable to tasks demanding conceptual understanding and extended reasoning across diverse mathematical contexts. This underscores the need for benchmarks with more complex, context-rich problems to better differentiate model capabilities beyond the observed ceiling effects.

There are some limitations that moderate these results. The relatively small dataset of 139 questions, while representative of the UNT exam format, may not fully capture the breadth of mathematical problem types. The benchmarks reveal ceiling effects for top-tier models like Deepseek, Qwen, Gemini, and o1, which scored 97.8–100% across all difficulty levels and topics in the zero-shot setting, suggesting the test may be too easy to distinguish their capabilities. This is likely due to limited question complexity, overlap with training data, and the absence of visual or diagram-based problems. Such high scores obscure differences in reasoning strategies, making it unclear whether, for example, o1's perfect accuracy reflects deeper efficiency than that of its peers. To better assess mathematical ability, future benchmarks should include more advanced and novel problems, incorporate metrics beyond accuracy (e.g., reasoning steps, error types), and test on diverse datasets—including Kazakh-language UNT items or visually based questions—to push models beyond their current performance ceiling and reveal more nuanced distinctions in their reasoning abilities. The perfect zero-shot result for the o1 model precluded it from hybrid and multi-agent testing, so its ability to improve further was not explored. Additionally, computational demands of hybrid and multi-agent approaches may be difficult to implement on a large scale within schooling. It should also be noted that the evaluations were conducted on specific snapshot versions of the LLMs. As these models continue to evolve with new releases, their performance characteristics may change, potentially affecting the replicability or relevance of the current findings. Finally, focusing on accuracy as a metric may overlook qualitative variations in model reasoning or error distributions, which may provide more illuminating information regarding their strengths. This study focused on Russian-language UNT questions, limiting insights into LLM performance on Kazakh-language versions, which are equally critical in Kazakhstan's bilingual context. Additionally, the simulated multi-agent refinement framework's external validity is limited, as all agents were simulated by the same LLM, potentially introducing confirmation bias in revisions. While the API-based approach ensures independent role processing, the lack of diverse models or human agents may limit error detection; future work should explore multi-model or human-agent frameworks to enhance robustness. Furthermore, the exclusion of 11 geometry problems requiring visual understanding may limit the assessment of LLMs' geometric reasoning capabilities, as our conclusions primarily apply to text-based mathematical problems.

Subsequent research should validate these findings across diverse mathematical datasets and standardized assessments to assess the applicability of hybrid and multi-agent techniques. Analysis of qualitative aspects, e.g., error patterns or patterns of reasoning, can further elucidate LLM abilities. Including metrics such as inference time or resource usage would help determine the practicality of these methods of augmentation. Further improvement of LLMs may be achieved by examining other symbolic tools or other multi-agent technologies, particularly for less capable models at baseline. Future work should also evaluate LLM performance on Kazakh-language UNT questions to ensure equitable applicability in Kazakhstan's multilingual educational system. Additionally, analyzing specific questions where models err could reveal limitations in their mathematical reasoning and guide targeted improvements.

## 6 Conclusion

Our findings indicate that top-performing LLMs like Deepseek, Qwen, Gemini, and o1 excel in mathematical reasoning in zero-shot settings, while hybrid approaches involving SymPy and multi-agent optimization considerably improve the performance of Claude. These augmentation techniques especially provide valuable outcomes for engaged problems and challenging mathematics domains like algebra and geometry, which are included in the UNT math test. For Kazakhstani students' learning, these findings suggest that student performance on the UNT could be improved by including sophisticated LLMs with symbolic reasoning or multi-agent systems in preparatory content. This content could facilitate adaptive learning, address mathematical gaps, and enhance readiness for university entry overall, to create a fairer and more effective system of education in Kazakhstan. As LLMs continue to develop, their implementation within educational platforms has the potential to revolutionize preparing students for demanding tests such as the UNT.

## Data Availability

The raw data supporting the conclusions of this article will be made available by the authors, without undue reservation.
